# Using candidacy theory to explore unemployed over-50s perceptions of suitability of a welfare to work programme: A longitudinal qualitative study

**DOI:** 10.1111/spol.12644

**Published:** 2021-07

**Authors:** Joanne Neary, Srinivasa V. Katikireddi, Ronald W. McQuaid, Ewan B. Macdonald, Hilary Thomson

**Affiliations:** 1 General Practice and Primary Care, Institute of Health and Wellbeing, University of Glasgow, Glasgow, UK; 2 MRC/CSO Social and Public Health Sciences Unit, Institute of Health and Wellbeing, University of Glasgow, Glasgow, UK; 3 Management, Work and Organisation, University of Stirling, Stirling, UK; 4 Public Health, Institute of Health and Wellbeing, University of Glasgow, Glasgow, UK

**Keywords:** longitudinal, older people, qualitative, unemployment, welfare to work

## Abstract

Welfare to work interventions seek to move out-of-work individuals from claiming unemployment benefits towards paid work. However, previous research has highlighted that for over-50s, particularly those with chronic health conditions, participation in such activities are less likely to result in a return to work. Using longitudinal semi-structured interviews, we followed 26 over-50s during their experience of a mandated welfare to work intervention (the Work Programme) in the United Kingdom. Focusing on their perception of suitability, we utilise and adapt Candidacy Theory to explore how previous experiences of work, health, and interaction with staff (both in the intervention, and with healthcare practitioners) influence these perceptions. Despite many participants acknowledging the benefit of work, many described a pessimism regarding their own ability to return to work in the future, and therefore their lack of suitability for this intervention. This was particularly felt by those with chronic health conditions, who reflected on difficulties with managing their conditions (e.g., attending appointments, adhering to treatment regimens). By adapting Candidacy Theory, we highlighted the ways that mandatory intervention was navigated by all the participants, and how some discussed attempts to remove themselves from this intervention. We also discuss the role played by decision makers such as employment-support staff and healthcare practitioners in supporting or contesting these feelings. Findings suggest that greater effort is required by policy makers to understand the lived experience of chronic illness in terms of ability to RTW, and the importance of inter-agency work in shaping perceptions of those involved.

## 1 Introduction

The UK, similar to other OECD countries, has an ageing population. Despite this demographic shift, it is not reflected in the participation rates of older workers in the labour market. In the United Kingdom, 81.9% of 50–54 year olds are in work compared to 49.5% of those 60–64 (DWP, 2015). This decline in labour market participation reflects both voluntary and involuntary exits, including retirement, as well as redundancy and long-term sickness leading to job-loss. This is a particularly vulnerable hidden population as, after 50, it is also more difficult to return to work (RTW) for a range of personal and organisational reasons. These include ongoing health concerns, outdated skill-sets, and experience of ageism in recruitment (Beatty and Fothergill, 2002, Neary et al., 2019), therefore risking extended periods of unemployment. These extended periods of unemployment leave older people at higher risk of poor physical and mental health (Whitley and Popham, 2017). There are relatively few policies which are tailored to supporting unemployed older people to RTW after a long period of unemployment, with the majority of targeted interventions focusing on retaining older people in work (Hamblin, 2010, Wübbeke, 2011). Unemployed older people claiming unemployment-related benefits are typically assigned similar interventions as their younger working-age counterparts, although they experience poorer RTW outcomes (Brown et al., 2017, Meager et al., 2014). To date, little is known about how older unemployed people perceive the suitability of these interventions to address their needs.

Haikkola (2018) used a broad governmentality approach arguing that while the implementation of activation policies may involve liberal ideas, such as self-governing individuals, they also involve governing the individual’s time and behaviour. Other researchers considered unemployed recipients’ views in relation to frontline workers, or street-level bureaucrats, (e.g., van Engen et al., 2019) or organisations, their networks and local communities (McQuaid, 2010). The current paper suggests that candidacy theory presents a useful framework to help understanding recipient’s changing perspectives and how they negotiate their eligibility and suitability for specific activation programmes based on their lived experienced, identities, and interactions with relevant institutions.

This paper specifically examines the perceptions of older people towards one of these interventions, the Work Programme (WP). At the time of study, the Work Programme was the largest welfare-to-work intervention in the United Kingdom. It focuses on their perceptions of their suitability for the intervention, and how these perceptions were informed by experiences of health, ageing, previous employment, and how these were mediated by interactions with WP staff. Using candidacy theory as our conceptual framework, we explore how individuals navigate the intervention. First, we provide a background of the policy context that frames our study.

### 1.1 Policy context: U.K. Unemployment policies

Across many OECD countries there has been a shift in welfare policy, whereby entitlements to social security has been tied to participation in mandatory “labour market activation” activities (Heidenreich and Rice, 2016); together with increasingly complex ways of trying to provide personalised and effective support (European Commission, 2019). In the United Kingdom and elsewhere over the last two decades there has been “creeping conditionality” (Dwyer, 2004) reflecting both the additional tasks required to be fulfilled by claimants, and the populations who are affected (e.g., lone parents, disabled people).

The assessment of unemployment benefits has also altered, with Invalidity Benefit and Severe Disablement Allowance being replaced with Incapacity Benefit (IB) in 1995 by the Conservative Government. IB had more restrictive criteria, which reduced the number of people eligible to claim (McKeever, 2000). In 2008, the Labour Government replaced IB with Employment Support Allowance (ESA), which had another conditionality attached: claimants had to participate in a medical test (the Work Capability Assessment, hereafter WCA) which scored a person’s capability to work (DWP, 2007). The assessment predominately focuses on physical functioning, where individuals scored points against a series of functional descriptors (Harrington, 2010) to find if they were fit to RTW. At the time, the UK Government’s Department for Work and Pensions (DWP) suggested this was “part of the drive to end sick-note Britain” (DWP, 2007). The Conservative/Liberal Coalition Government, 2010–2015, combined ESA with Job-seekers Allowance (JSA), the unemployment benefit given to those without health conditions, to create Universal Credit (UC), which combined Housing Benefit, various unemployment benefits and Child Tax credit into one benefit. Dwyer and Wright (2014) describe UC as “intensified, personalised and extended conditionality.”

Critics of this approach highlight that: these interventions focused too much on individual aspects (e.g., motivation and skills of the individual) rather than on the structural causes of unemployment; are poorly suited to meeting the needs of the changing labour market, for example, zero hours contracts (Mattheys et al., 2018); risks “creaming” of those nearest the labour market and “parking” of those with complex needs by unemployment agencies (Carter and Whitworth, 2015); and ignoring the overlapping issues present in the lives of benefit claimants (Garthwaite, 2015;Wright, 2016).

#### 1.1.1 The Work Programme

The Work Programme (WP), introduced by the UK Coalition Government in 2011, focused on long-termed (mainly 1 year+) unemployed and other groups facing difficulties in returning to work. Similar to other countries, such as United States, Australia and Germany, the U.K. Government subcontracted many employment services to private, and in some cases third sector, contractors. 18 providers were contracted to manage employment services in 40 “contract areas” across the United Kingdom and could choose to deliver the service themselves or subcontract to specialist agencies. The WPdE was a “black box” intervention, which allowed flexibility in how each provider could deliver the intervention, although the majority followed a similar delivery model (Foster et al., 2014). WP providers were paid by results—mainly based on the individuals retaining employment for a pre-determined time and their closeness to the labour market.

Evaluations of the WP highlight that certain subgroups are more likely to experience a job start than others (Meager et al., 2014). For example, individuals claiming JSA are more likely than those claiming ESA to experience a job start, 18–24 year olds are more likely than people over-50, and people with more recent work experience more likely than those with long-term unemployment. The population who leave the two-year WP without a job-start, were more likely to be male, older than 55, have a disability or health condition, have low/no qualifications, and have experienced limited employment prior to joining the WP (Meager et al., 2014, Brown et al., 2017). Most literature on the WP focuses on the wellbeing of individuals after entering paid work, with relatively little focused on experiences of participation in the intervention itself (Carter and Whitworth, 2015). Given that older people are less likely to RTW, it is of interest to explore their perceptions of suitability of WP to support them.

### 1.2 Theoretical framework: Candidacy theory

Candidacy theory captures the idea that how individuals view their suitability for interventions and services is socially constructed. By socially constructed, we refer to the ways in which individuals’ perceptions of eligibility and suitability are seen to be the product of negotiations between individuals’ lived experience, identity, and interactions with institutional actors. Initially, candidacy theory (Dixon-Woods et al., 2006) explored inequalities in accessing health care, and how factors such as socio-economic status, gender, and ethnicity might explain these differences. The theory is described as a set of stages including locating care, demonstrating condition to professionals, and the response by professionals (either confirming or denying) which may in turn be informed by meso- and macro-level forces (such as institutional or policy factors).

Most of the work conducted using candidacy theory explored healthcare utilisation and lay epidemiology. For example, Emslie et al. (2001) explored women’s perceptions of coronary heart disease, finding that they believed likely candidates were perceived to be men, meaning they were less likely to believe themselves to be at risk while Purcell et al. (2014) described the tension between conflicting candidacies with regards to abortion (being a candidate for pregnancy or abortion).

In the social sciences, Mackenzie et al. (2012) explored the utility of candidacy when reviewing domestic abuse, higher education and environmental services, finding a high level of congruence between candidacy and utilisation of services, exacerbated by gender, experience of poverty and ethnicity. This suggests it is a useful model for acknowledging the role of professionals in sifting potential users at the point of access, and of the front-line worker in making these decisions (Tummers and Bekkers, 2014).

While previous work has focused on help-seeking behaviour and “claiming candidacy,” we are interested in utilising Dixon-Woods et al. (2006) stages of candidacy (see Figure 1), to ask how candidacy can be understood in scenarios where the candidate was pre-selected by the organisation running the intervention, and candidacy was enforced or mandated.

Previous work on candidacy theory notes that eligibility criteria of “being a candidate” is set by policy makers, with the decision-making process conducted by front-line staff. This comment is also true in the context of our study, as policy makers determine who is required to attend the WP, and how health conditions are “scored” in the WCA (Leitchfield, 2013).

For this paper, we focus on those individuals who have experienced the WCA as it represents an important “adjudication” in their journey: whether their health status is judged severe enough to stop job-seeking, or whether they are seen as candidates for the WP. For participants in the study, “passing” the WCA may mean they still experience chronic health conditions, but that these are viewed by an assessor as not posing a barrier to RTW. Hence, this becomes the first step in our mandated candidacy model: organisations determining whether you fit the criteria to be a candidate for intervention.

In exploring the dynamic journey of individuals during the WP, we are also interested in whether the participants have identified other candidacies that either support or reject their current experience in the WP, and how they navigate this. Previous studies have highlighted some instances where individuals assert their *lack of fit* with healthcare interventions informed by their lay understandings of the “ideal candidate” (Hunt et al., 2001), leading to resistance to offers of medication (Britten et al., 2004). For those who believe they are not candidates, and contest their presence in the intervention, we also examine how this was managed in the context of a mandated intervention.

The next section presents the methods followed by our findings and then a discussion and conclusions.

## 2 Methods

The data analysed and presented in this paper were collected as the qualitative component of a larger longitudinal mixed methods study looking at RTW in over-50s, “Supporting Older People into Employment” (SOPIE) (Brown et al., 2015, Neary et al., 2019). The current paper analyses qualitative longitudinal data gathered over the period 2015–2017. The fieldwork period reflected the duration of participants’ engagement with the 2-year WP. Wave one recruited participants 3 to 9 months into the intervention, and wave two re-interviewed them between 18 and 24 months. The longitudinal nature of the fieldwork enabled us to capture how participants’ perceptions and experiences of the WP changed over time, and to explore their causes (Thomson and McLeod, 2015). The period between interviews was appropriate (given the 24 months length of the intervention) to trace changes in views during actual participation and before leaving the programme (after which point memories and perceptions might be influenced by subsequent experiences).

### 2.1 Recruitment

#### 2.1.1 Wave one (3–9 months participation in the WP)

Participants were sampled based on age (50–64 years), and duration of engagement in the WP (between 3 and 9 months). Both ESA and JSA claimants were recruited. Recruitment of participants to Wave one was conducted in two stages, as required by DWP. A description of the recruitment process can be found in the protocol paper (Brown et al., 2015).

Of the 750 who were first contacted, 120 agreed for the research team to contact them. Additional information was posted to them to ensure informed consent. After a follow-up phone call, individuals were asked if they were interested in participating. 26 agreed. Others declined to participate, were unavailable, telephone numbers were incorrect, or did not show up at agreed meeting time. Diversity in terms of gender, claimant group (JSA and ESA), and time unemployed, was sought and achieved, but importantly we did not attempt to achieve a representative sample of WP participants for this exploratory research.

#### 2.1.2 Wave two (18–24 months participation in the WP)

All Wave one participants (n = 26) were sent a letter in the months preceding the wave two interviews. The letter informed participants that JN would be in contact to arrange a follow-up interview with them, and that the purpose of the second interview was to explore any changes that occurred in their lives over the interim period between waves one and two. The letter also highlighted that if nothing had changed, or if they had left the WP, the team were still interested in talking to them. Difficulties were experienced in re-contacting participants. Seven (n = 7/26) participants’ phone numbers were no longer in service, and therefore could not be contacted. A further four participants initially agreed to participate but did not respond to further calls. Fifteen participants completed the second interview. Demographic information regarding participants can be found in appendix one.

### 2.2 Interviews

All Wave one interviews were conducted face-to-face; with just under half of Wave two interviews were conducted via the telephone. Interviews lasted between 30 and 90 min. Telephone interviews were requested by participants who could not meet in person for a variety of reasons (including health or unpredictable shift patterns at work). Given the rapport built in wave one, JN did not find the data collected via telephone interview was of a diminished quality.

Wave one interviews followed a semi-structured interview script (see appendix two). Wave two was more participant-led, beginning with an open-ended question “how have the last 12 months been for you.” The question was structured in such a way to allow the participants to direct the conversation to the most salient aspects of their lives. All interviews were recorded on an encrypted Dictaphone.

Consent was an ongoing process during interview, with opportunities to “pause” interview, skip questions, or stop the recorder given. Reasons for pausing included having an emotional response to a question, moving around due to sore joints, or more everyday activities like answering personal calls.

### 2.3 Analysis

Interviews were transcribed and uploaded to a qualitative data organisation and analysis package (QSR NVivo 10). Transcripts were analysed using thematic analysis (Braun and Clarke, 2006), adopting a social constructionist approach (Sharf and Vanderford, 2003). We were interested in how participants’ perceptions and decisions regarding eligibility and suitability of the WP were informed by the wider contexts of their life, such as health and age.

JN coded all data, with SVK and HT conducting secondary analysis on 25% of data. Wave one data were first coded looking for initial discussions of WP experiences. This included their initial referral to the WP, interactions with advisor(s), and experience of workshops. Underpinning these experiences was a narrative surrounding suitability of these services for them. In early analysis, JN coded the theme of “suitability” to reflect participants’ narratives regarding how they felt their own needs would be met by the WP. After discussion with all authors on the utility of candidacy as an analytical framework, the theme of “suitability” was expanded to also include participant interactions with staff members, their own perception of health, and barriers to work.

Wave two data were coded by JN and RM, and explored instances of “change” in terms of biography, health, and occupation, and also the interaction between micro and macro level contexts, particularly whether changes in the participant’s personal life (Lewis, 2007) impacted on their participation in the WP. In this paper, we highlight those data where participants’ belief in their candidacy had changed in the 12 months between interviews, specifically whether it had remained the same, or whether another candidacy had emerged (e.g., “retirement”).

## 3 Results

All participants interviewed were working-age candidates in the Work Programme claiming state support (JSA or ESA), and had passed the WCA (the “adjudication” stage of candidacy framework, see below). Our research illustrated that understandings of candidacy were more complex and intersectional, with narratives of suitability informed by interactions between age, gender, health, skills and education level, and location.

The results follow a framework shaped by the stages of candidacy theory, acknowledging the similarities and differences therein. One key area of difference is that the WP requires individuals to participate in programmes of work and training, failure to do so may lead to financial sanctions. We therefore explore how participants who disagree with the adjudication, seek a second opinion and may seek alternative candidacy while also complying with WP activities.

### 3.1 First stage: Adjudication

For participants on JSA, adjudication was through the government’s DWP that manages unemployment benefits. These claimants would be alerted that they were to be reassigned to WP. Those claiming ESA attended a WCA, which was often viewed in a negative light:

“A person that does a quick medical assessment for five minutes doesn’t know you at all. You could be having a really good day that day when you go into that assessment an ‘they’ll say ‘There’s nothing wrong with you’. But that could be a really good day out of fourteen totally crappy ones” (Steve, 60–64, ESA, W1)

Similar to previous studies (Barr et al., 2016a, Baumberg et al., 2015), participants discussed a frustration with the assessment process, particularly if their condition was chronic or fluctuating in nature. This also related to their experience of the simplistic “physical” tests of the assessment:

[they asked] like “how do you lift spoons and cups.” That’s really degrading, its being in play school. I had to pull chairs out and sit doon [down] and then stand up on these steps and sit up on a stood. (Mike, 50–54, ESA, W1)

They suggested that these skills did not reflect the things they did have difficulties with, and therefore created a false narrative about how “fit for work” they were. This frustration also reflected the lack of WCA acknowledgement of treatment plans they had with their GP (general practitioner, family doctor) or other medical professional, who were seen as having a more nuanced long-term understanding of their capacities. As a result, many felt their health condition was not fully understood, and their assessed capacities to RTW were not correct. In turn, this experience was an important element of some participants’ perceptions that they were not candidates for the WP.

### 3.2 Second stage: Affirmation or contestation

For those who had a negative experience of the WCA, health-related issues were seen as only part of the reason for their contestation:

“I won’t be able tae [to] do anything in the past that I have done, you know? So I’m thinking about going, retraining. [I’m] struggling wi’, you know, the limits, the limitations on the sort o’ work that I’m qualified to do. I mean, I’m null an’ void, you know” (Steve; 50–54, ESA, W1)

Steve had previously worked in a physically demanding job but, after major heart trouble, was made unemployed for the first time. He described a conflict in his candidacy, between wanting to go back to work, but knowing that he could not return to employment through reasons of health and skills. He described the WCA as being limited in scope, it stated he could work but did not suggest where he could work or what he could retrain as. Instead, he felt *“null and void,”* so thinking the work-focused WP was unsuitable currently. The conflict between “being a worker” and “being sick” was also described by others who had previously been long-term employment:

“I have severe depression, if I’m in crowds I have panic attacks, I walk with two crutches because of my scoliosis, and I’ve got arthritis in my spine and in my hips…there’s not a lot I can do because it is too sore… [but] not working is boring, when you’ve worked constantly and then all of a sudden you’re not working, I miss seeing people. I can go days without seeing anybody” (Janet, 60–64, ESA, W1)

Similar to Steve, this current period was Janet’s longest period of unemployment. She described missing work and the social element, and the impact this isolation had on her mental health. However, there was a mismatch between her previous experience in skilled employment and the limits imposed by her physical and mental health conditions. The WCA showed that she could work, but not what she could work as, or how to access training.

For others, their rejection of candidacy was informed by age, health, and gender:

“When I went onto the WP, I didn’t know how long it would last, I didn’t realise it was over two years. I’ll be sixty-two when I finish, you know? Why are they doing this with older people…they’d be better doing it for younger people who have got a full lifetime to work” (Julie, 60–64, ESA, W1)

For those nearing retirement age, several described a desire to be *“left alone”* rather than receive targeted support to RTW, particularly for those women who were affected by the announcement that U.K. women’s pensionable age was moving from 60 to 65. This macro-level policy change with regards to who is “eligible” was viewed as incompatible with the plans and capabilities of the older women interviewed. When describing their ideal next step, they discussed their desire to retire to provide support for their adult children, or to take care of grandchildren.

Participants also discussed who they felt the “ideal” candidate was, often in their discussion of how far away from the “ideal” they were. For these participants, candidacy in the WP was equated to being “fit” and “healthy” and being able to re-join a flexible and competitive workplace. Through their experience of ill-health, and managing their conditions, they felt this was not in line with their own situation and needs. However, they used a similar strategy to distance themselves from the “typical”/”ideal” unemployed person that is often presented in the media. They reiterated that they wanted to work, and that they saw the benefits of working in terms of health. In this instance, we see that they still portray themselves as “workers”, despite currently being unable to do so. Again we see multiple candidacies at play: to be “deserving” of being unemployed rather than going through the Work Programme and to be “genuinely” sick rather than those portrayed in media. However, not all participants disagreed with their candidacy, agreeing it was a “good fit”:

“[My advisor] explained what the WP was, and I was quite happy about it. I thought it was something that would kinda help me” (Marie, 55–59, ESA, W1)

For some, like Marie, who had previously participated in other RTW interventions, they believed the WP would offer similar advice. For those who felt ready to RTW, the WP fitted in with their perception of self. Embedded in this were reflections of being an “active jobseeker” and a “worker.” Similar language was used by both those rejecting and accepting candidacy: they perceived themselves as fit, flexible in their expectations of work, so they were able to take on a variety of jobs. In their discussions of the WP, they also reflected on the health benefits of work, reiterating the discourse embedded in work activation policy—that work is good for your health. Four participants felt positive about the WP, three claimed of whom claimed JSA.

### 3.3 Third stage: Navigation and appearing

Navigation was the longest stage in the participants’ journey, often lasting the full duration of their 2 years engagement with the WP. By navigation, we refer to the work done by participants in complying or contesting with the WP. For both groups, this work included attending appointments and workshops, but for those contesting their candidacy, there were additional resources to seek out and access.

For those who affirmed their candidacy, navigation of services included working with the WP advisor to attend workshops and participate in job-finding activities:

“the individual that is taking care of me there…she’s wonderful, you know? And she’s got me the odd job here and there, albeit always temporary, you know? But she does her best, you know, for what’s out there (Javi, 50–54, JSA, W1)

One piece of work required for those who affirmed their candidacy, was that of accepting the limitations of the job market. They were not rejecting short-term contracts or criticising their advisor for not doing more, but rather promoted an image of themselves as being happy to comply and understand the labour market, including the limitations of the job opportunities it offered.

For those who contested their candidacy, navigation involved attending WP appointments but also doing additional “work” in communicating, asserting, and finding agreement for, their contested candidacy with professionals, both within the WP and externally. For those who believed they had been wrongly assessed by the WCA, their GP was often a supportive figure in their contested candidacy:

“When you go to the job centre and still have to sign on, and they say ‘why haven’t you got a job yet’, but my doctor is telling me I’m not fit to work, and so is the hospital…so who do you listen to?” (Ian, 50–54, ESA, W1)

This conflict between professionals, particularly how health is understood across the different organisations, led some of the participants to describe being unsure about which professional to trust. This potential conflict between healthcare professional’s focus on the individual’s health needs and pressures from other professions to get people back into work was also an issue in earlier activation policies (Lindsay et al., 2007). This had an impact on their belief regarding their ability to RTW. Knowing who to trust was often based on qualifications, and existing relationships, particularly where participants had chronic conditions requiring multiple appointments in healthcare settings.

In addition to healthcare providers, at times, participants described their WP advisor as supporting their contested candidacy:

“The person I’ve been working with actually said to me ‘you shouldn’t be here’ as if to say ‘I don’t know where we’re going to get you into work’” (Richard, 55-59, ESA, W1)

This was described more by those with more visible physical health conditions who described receiving “sympathetic treatment” by advisors. This included advisors helping them apply for the ESA “Support Group” which would end their mandated participation in the WP, suggesting wellbeing, rather than work-focused, classes, and having informal conversations with the individuals rather than work focused discussions.

For those who contested with their candidacy for the WP, there was a complex navigation where they were required to present as a “good” candidate (job searching, participation in training, attending appointments) as failure to do so would risk financial penalties. This led to a situation where some participants applied for jobs they would not succeed in, to ensure they were perceived as actively seeking work. They did not see this as personally beneficial, but rather did so to keep the organisation “happy,” and therefore avoid penalty.

For one participant, they also described “playing the system” to ensure they were seen as less employable than they were in reality. This involved choosing not to disclose they could use computers, playing on a stereotype of older people and computer illiteracy. In wave one, they described this meant they were required to look for fewer jobs, and the jobs they applied for could not have IT skills. In wave two, they described this ploy as *“backfiring”* as their advisor requested they attend weeks of basic IT skill-focused training. The participant made the decision to disclose their IT skill level, to avoid continuing with the *“boring”* training. This resulted in their job-searches being broader, although at time of interview, had not resulted in a job start.

### 3.4 Fourth stage: Re-affirmation and re-adjudication

Given the longitudinal nature of the qualitative study, we were able to follow participants during their two-year engagement with the WP. As discussed above, this enabled us to explore the journey of candidacy, asking whether people’s perception of mandated candidacy changed after experiencing the intervention. We also explored how the success or failure to achieve a job start impacted on their perception of suitability (see appendix three for RTW rates). This last stage reflects on the wave two follow-up.

For some, their initial optimism for their candidacy in the WP had been tested by their lack of re-entry into employment:

“I have just kind of given up with it. At my age and what I’ve got, and I’ve tried and tried and tried, and I’ve not got anything, and [WP provider] have not gotten me anything…I don’t know what else you could do” (Beth, 60–64, ESA, W2)

While initially wary of the WP, and the utility of the intervention to support her to RTW, Beth participated fully in the intervention, and was an active job seeker. Beth described attending workshops, working on her CV, and applying for a range of jobs. However, she lived in an area with a depressed local economy that had poor local transport links which created difficulties in finding work. She described behaviours as fitting with the “active” jobseeker model but became discouraged by her lack of progress. This affirmed to her that the WP was not for her, and that she would not be able to RTW.

Wave two also found a group of individuals who, in the interim period, had found an alternative candidacy, other than work or retirement. Three women at wave one described experiences of chronic health conditions, and a pessimism regarding returning to work. At wave two they described the positive impact volunteering had on their life. While experiences of chronic pain were still central to their experience of barriers to work, they had found a work-place that was flexible in their attitude towards their conditions. When discussing paid work, they suggested that employers would not take such a flexible attitude, and instead may be more critical of repeated absences. While their work was not paid, both described experiencing the same types of psycho-social benefits as paid employment:

“It gets me out, gives me a reason tae [to] go out. I do it on a Saturday morning for three hours, ten until one, but I thoroughly enjoy it… All the volunteers, we’ve nearly all got [a health condition], but I’m the worst, I’m the only one who walks with two crutches, but they’ve all got something wrong.” (Janet, 60–64, ESA, W2)

Volunteering gave some of the same benefits of work, such as a sense of purpose, and social contact. Given that at wave one, they all described feelings of social isolation, this was an important change for them. Also important for them was that all the volunteer staff they worked with had health conditions too, so there was a sense of community of being among people “like them.”

For others, their experience of chronic health conditions led them to attend a re-assessment at the WCA centre, hoping that they would be able to accrue enough “points” to leave work-activation interventions such as the WP:

“I had to go for the appeal, and it has been going on since then. The appeal finally came around, so all that time from last June right through, my health was so bad, really bad. Mentally, physically and emotionally” (Kathleen, 50–54, ESA, W2)

As Kathleen’s condition involved both a mental and a fluctuating physical health condition, she was concerned that the assessor may not believe her alternative candidacy for the “Support Group.” It was also exacerbated by stress, and she believed that the wait between the initial assessment and the appeal had triggered her symptoms. However, she believed that once she was able to attend to her health, she would seek self-employment opportunities to enable a flexible working pattern that fitted with her health needs.

## 4 Discussion

### 4.1 Applying candidacy theory to a mandated intervention

Welfare to work interventions seek to move out-of-work individuals from claiming unemployment benefits and towards paid work. However, previous research has highlighted that for over-50s, participation in such interventions is less likely to result in a return to work. This paper has highlighted the ways in which candidacy theory could be applied to the context of work activation interventions. While previous studies utilising candidacy theory explored experiences of individuals voluntarily navigating services in order to prove themselves as candidates for an intervention, we explored *mandated* candidacy where an intervention pre-selects individuals as candidates. In doing so, we adapted the Dixon-Woods model to reflect the experiences of our participants (see Figure 2).

Our analysis of participants’ perceptions of the suitability of this intervention was informed by age, gender, skills, and previous employment, and health. At times, these elements clustered together, with age and gender informing participants’ belief that they were a candidate for retirement rather than a work activation scheme. Health, age, and skills clustered where participants previously held one skilled job for a significant amount of time, and through ill-health felt they were unable to return to this career. It left participants feeling conflicted regarding RTW. While many spoke about their previous work histories, and the benefit of work to reduce feelings of loneliness and to give purpose, many felt they were not currently able to participate in the workforce. Following participants over 2 years, the duration of WP engagement, showed that some of this group were able to gain some benefits associated with paid employment through volunteering. However, this was not viewed as a “success” for the WP, as it was unpaid.

The use of longitudinal qualitative methods in this study also enabled us to explore the “journey” of candidacy in the intervention. By going beyond the initial impressions, we were able to explore how participants navigated the intervention, and how different interactions affected their perceptions. Also, examining how their own identity and beliefs regarding capacity shaped their understanding of suitability, and interaction with staff influenced these.

Similar to previous work regarding candidacy theory, we saw the “work” underpinning being a “good candidate,” and the importance of presenting as such in a mandated intervention. Some discussed worries of sanctions, or what would happen if they did not attend meetings or find enough work to apply for. Of interest was how many took on additional “work” to pursue an alternative candidacy, seen in those who contested their candidacy for the WP. This led to a dual workload, having to attend the WP, look for work, attend interviews, while also gathering information and support from individuals like GPs to support their “sick” candidacy. Similar to the work of Macdonald et al (2016), asserting this alternative candidacy was not straightforward, and left particiants constantly reasserting their claim, experiencing uncertainty and requiring understanding, or protection, of stakeholders. The work of being a “good” job-seeker, and also of seeking to leave the WP was done in conjunction with treatment burden associated with their condition: daily routine of taking pills, ointments, attending GP or other health professionals, and adapting lifestyles.

We also found evidence of candidacy being the product of negotiations between individuals’ lived experience, identity, and interactions with institutional actors, in particular the significant role of their healthcare provider (e.g., GP) to support the case for their contested candidacy. For some, this proved fruitful, as they challenged the WCA result and moved to the Support Group. For others, this navigation was ongoing. Turning to healthcare providers to support the rejection of the result of the WCA creates a conflict between employability services and primary healthcare, with the tension residing on the question “who is fit for work, and what does this mean.” For others, the key institutional actor was the WP advisor they worked with, who was sometimes seen as being sympathetic and agreeing with their contested candidacy. This is similar to Kaufman’s (2019) description of some advisors “parking” clients to protect them from unwanted or unhelpful interference.

The findings suggest that greater effort is required by employability services to understand how people feel about mandated candidacy for interventions. More work is also needed to understand how people utilise resources to inform their decisions regarding candidacy for these interventions; particularly the important role of GPs/health professionals in providing an alternative, perhaps more trusted, judgement regarding the capacity of RTW for individuals with chronic health conditions. Also, while volunteering is not seen as a valid outcome for the WP, our findings show multiple motivations for volunteering including social interaction, doing useful work and getting work experience (Griep et al., 2015; Waikayi et al., 2012), and that engaging in such work had a positive impact on people’s self-esteem and confidence. Our work highlights the potential contestation between professionals, as well as patient-professional relationships, that is possible in assessments of candidacy.

### 4.2 Strengths and limitations

Our work has several limitations and strengths. Like other longitudinal studies, there was a drop-off in participation between wave one and two, therefore not all stories could be followed up. This was particularly an issue where participants described being close to RTW in wave one. The study was the two-stage opt-in recruitment required by the DWP prior to accessing participants engaging in their WP, leading, we believe, to the large drop-off in potential participants. The sample was not representative or generalizable to WP or ESA clients, or even of those who might contest their candidacy, but rather sought to explore key issues.

The use of candidacy theory enabled a deeper discussion of older people’s experience of the WP, beyond initial “barriers to work” discussions, and highlighted the role of health, identity, gender, and age on perceptions of suitability. Previous work has highlighted that over-50s are among the hardest to help in relation to RTW, and candidacy theory enabled us to take a closer look at their perceptions of suitability of the intervention to their circumstances. Also, the longitudinal design of the study enabled us to look at the entire duration of the WP, rather than taking a snapshot. By using repeat interviews, we were able to look closely at the negotiations of the participants, and whether changing life events and relationships influenced their perception of candidacy.

### 4.3 Conclusions

The perception of health in relation to the ability to return to work is complex. On one hand, all participants in this study were assessed by a professional as being able to participate in the Work Programme. On the other, their lived experience of health, and interaction with primary healthcare providers suggested many should not RTW. This conflict lies at the heart of our study. Our study used candidacy theory to explore how participants made sense of this conflict, where one stakeholder group mandates candidacy for an intervention, while another suggests an alternative candidacy.

There are implications for policy makers and health professionals in terms of how they support older people in returning to work and how they implement mandatory health assessments such as the WCA. While it is important to ensure that all unemployed populations should be supported to return to work, the criticism of the WCA in pushing, often vulnerable, people into situations where they are being asked to look for work, while also managing complex health conditions, does not always appear fair or effective. Rather than supporting positive employment outcomes (Barr et al., 2016b), policies such as this may instead add to negative mental health experiences.

Finally, we found where individuals disagree with their candidacy, many engaged in *additional* “work,” alongside the job seeking activities mandated by the intervention, to try to protect themselves (to remove themselves from the intervention, or to alleviate some of the conditions of the intervention). These additional activities were done while also being mindful of the need to look like an “active jobseeker” while negotiating an alternative pathway. Therefore, while work may be good for your health, for many the experience of labour market activation was not.

## Supplementary Material

Appendix

## Figures and Tables

**Figure 1:** stages of candidacy (taken from Dixon-Woods et al., 2006) First stage: Identification: how people recognise their symptoms as needing medical intervention Second stage: Navigation: the “work” people must to do to use services and access resources Third stage: Permeability of services: the ease to which people can use services (and the qualifiers required to access services) Fourth stage: Appearing: people asserting their claim to candidacy for medical intervention (involving skill to articulate their issues, and align these with criteria) Fifth stage: Adjudication: professional judgement about individual’s bid of candidacy

**Figure 2:** stages of mandated candidacy (adapted from Dixon-Woods model) First stage: Adjudication: professional judgement about individual’s suitability (or eligibility) for intervention leading to mandated candidacy Second stage: Affirmation or contestation: Individual’s initial perception of ‘fit’ of intervention to address their needs. Leading to affirmed candidacy, or contested candidacy. Third stage: Navigation and appearing: The work done by individuals both in complying with the intervention, but also in asserting their contested candidacy (including seeking resources, other opinions, while also meeting mandated requirements) Fourth stage: Re-affirmation and re-adjudication: Individual’s perception after experiencing intervention. This stage also includes re-adjudication where candidacy was successfully contested.
