# Supplementary material for: Using candidacy theory to explore unemployed over-50s perceptions of suitability of a welfare to work programme: A longitudinal qualitative study
Source: Soc Policy Adm. Author manuscript; Available in PMC 2021 Nov 16. (PMC7611994; doi:10.1111/spol.12644)
Supplement: Appendix [file EMS137981-supplement-Appendix.pdf]

APPENDIX 1: DESCRIPTIVE DATA

	Category	(*n* = 26)
Gender	Female	14
	Male	12
Age	50–54	13
	55–59	7
	60–64	6
Benefit type	Job seekers allowance	10
	Employment support allowance	14
	Universal credit	2
Health condition	No	1
	Yes- mental health	7
	Yes- physical health	8
	Yes- both physical and mental health	10
Last occupation (based on standard occupational classification)	No employment history	1
	Elementary occupations	6
	Process/plant/machine operatives	3
	Sales and service occupations	1
	Caring and leisure	5
	Skilled trades	2
	Administration/secretarial	1
	Associate professional and technical	4
	Professional occupations	3
	Senior officials	0
Educational attainment	No qualifications	6
	Standard grade/o-level/leaving cert	4
	A-levels/Highers	1
	Vocational qualifications (inc. city and guilds)	3
	College level qualification	6
	University degree	6
Length of unemployment	< 12 months	5
	1–2 years	0
	2–5 years	15
	> 5 years	6

APPENDIX TWO: INTERVIEW SCHEDULE

How would you describe your physical health at the moment?

How would you describe your mental health?

Do you have any specific medical conditions?

Do you think your health just now has an effect on (a) your ability to socialise (b) your daily routine (c) your search for work?

What medications are you currently taking?

Do you see a doctor, GP, other medical professional?

Have they ever spoken to you about working?

Have you had experience of having your health assessed for the purpose of benefit entitlement?

What benefit are you currently claiming? How long have you been in receipt of this?

Do you receive any other financial support? (Eg PIP, housing benefit etc)

What was your last experience of paid employment? Where was it, how long were you there, what did you have to do in the job? How did you learn about this job?

When did you leave this job? What were the reasons behind this?

When you look back at your working life, would you say the majority has been employed, unemployed, caring for family, or a combination of the above?

Have you ever considered volunteering?

Do you think health and work are related? In what ways?

Have you experienced any positive or negative attitudes by employers to people over-50?

Do you think unemployed people who are over-50 would want to return to work?

What do you think are the main challenges of finding work for people over 50?

Do you have any concerns about returning to work?

APPENDIX THREE: RETURN TO WORK RATE AT WAVE TWO (BY GENDER, BENEFIT TYPE, AGE, YEARS UNEMPLOYED)

	Gender		Benefit type		Age			Time unemployed prior to WP			Total
	F	M	JSA	ESA	50–54	55–60	61+	<12 mths	1–3 years	4+ years	/
Unemployed at wave one/employed at wave two	1	2	3	0	2	1	0	1	1	1	3
Employed prior to wave two/ unemployed at wave two	2	0	2	0	1	1	0	0	1	1	2
Consistent unemployment at waves one and two	6	4	3	7	3	3	4	2	3	5	10
